# Ideal Cardiovascular Health Index and Its Determinants in a Rural South African Population

**DOI:** 10.5334/gh.801

**Published:** 2020-11-25

**Authors:** E. J. Ketelaar, A. G. Vos, N. G. Godijk, K. Scheuermaier, W. Devillé, H. Tempelman, R. A. Coutinho, W. D. F. Venter, D. E. Grobbee, K. Klipstein-Grobusch

**Affiliations:** 1 Julius Global Health, Julius Center for Health Sciences and Primary Care, University Medical Center Utrecht, Utrecht University, Utrecht, NL; 2 Ezintsha, Wits Reproductive Health and HIV Institute, Faculty of Health Sciences, University of the Witwatersrand, Johannesburg, ZA; 3 Brain Function Research Group, School of Physiology, Faculty of Health Sciences, University of the Witwatersrand, Johannesburg, ZA; 4 Ndlovu Care Group, Groblersdal, ZA; 5 Division of Epidemiology and Biostatistics, School of Public Health, Faculty of Health Sciences, University of the Witwatersrand, Johannesburg, ZA

**Keywords:** Ideal cardiovascular health index, cardiovascular disease, carotid intima-media thickness, socio-economic status, HIV, South Africa

## Abstract

**Background:**

The ideal cardiovascular health index (CVHI) is a measure to summarize cardiovascular (CV) health, and includes smoking, body-mass index, physical activity, blood pressure, glucose, total cholesterol, and diet.

**Objective:**

This study aimed to assess CV health using the CVHI and determinants on CV health in a rural African population, and correlate carotid intima-media thickness (CIMT), a surrogate marker for atherosclerosis, with CVHI.

**Methods:**

A cross-sectional analysis was performed on baseline data of the Ndlovu Cohort Study, located in rural South Africa. CVHI score (CVHIs) was calculated by the sum of favourable CVHI factors (range 0 to 7). Logistic regression was performed to examine the association of age, sex, HIV-status, education level, employment status, and income with good CV health (5–7 favourable health factors). Mean CIMT was displayed by poor, intermediate and good CV health.

**Results:**

The study included 1927 participants with a mean age of 38.7 years (SD ± 12.8). Of the factors contributing to the CVHI, glucose and total cholesterol scored best; diet least good. Average CVHIs for the population was 4.4 (SD ± 1.2) and 53% of the population had a good CV health. Determinants associated with good CV health were younger age, higher educational attainment, and HIV positivity. CVHIs showed good agreement with CIMT.

**Conclusion:**

CVHIs showed that more than half of the participants had a good CV health. Agreement between CVHIs and CIMT indicates potential use of CVHIs as a surrogate marker for CV risk. The study highlights the importance of education for health promotion; good CV health in HIV-positive participants may in part be attributed to more frequent health care contact and provision of chronic disease care.

**Highlights:**

- Good cardiovascular health (CVH) was observed in 53% of the study population.
- In global comparison, rural African study participants showed a good CVH score.
- HIV positivity was associated with a good CVH score.
- CVH score showed good agreement with carotid intima-media thickness.

## Introduction

Cardiovascular disease (CVD) is the number one cause of death globally, causing 31% of all global deaths in 2016 [1]. Approximately 80% of the CVD burden is carried by low- and middle-income countries (LMIC). Between 1990 and 2015 a decline in age-standardized CVD mortality was seen in all high-income countries and some middle-income countries, but this trend was not seen in most countries in sub-Saharan Africa (SSA) [2]. CVD related deaths increased in South Africa with 25.5% between 1990 and 2017, and was the number two cause of death, after sexual transmittable infections including human immunodeficiency virus (HIV) [3]. With increasingly improved management of infectious diseases such as HIV and tuberculosis, life expectancy is increasing and hence the rise in the occurrence of non-communicable diseases, including CVD.

The Ideal Cardiovascular Health Index (CVHI) is used by the American Heart Association (AHA) as a tool to summarize cardiovascular health (CVH), and amalgamate traditional risk factors for CVD including smoking, overweight, high glucose, high total cholesterol, high blood pressure, unhealthy diet, and physical inactivity [4]. Several studies have already reported the CVHI, however only two studies have investigated CVHI in SSA [5,6,7].

Socio-economic status (SES) has been associated with CVD [8,9]. However, studies on the association between SES and CVH in LMIC are scarce [10]. The additional role of HIV in CVD risk prediction has been established in recent years, but the mechanisms of CVD in regard to HIV viremia and the role of antiretroviral therapy (ART) remains poorly understood [11].

In the absence of longitudinal data, the CVHI may be a marker for subclinical atherosclerosis. To assess the relation between CVHI and subclinical atherosclerosis, carotid intima-media thickness (CIMT), a well-established marker for subclinical atherosclerosis, can be used. CIMT is an ultrasound-based measurement of the thickness of the intima and media layer, referring to the common carotid artery as utilized in this paper. An increase in vessel wall thickness has been related to an increased risk of CVD [12]. CVHI does not require the technical equipment and expertise as is needed to obtain CIMT, making it an attractive proxy substitute for CIMT measurements to estimate CVD risk.

This study aims to assess the CVHI of a rural South African population and to investigate determinants of the CVHI. In addition, CVHI was assessed as a surrogate marker for CVH as compared to CIMT.

## Methods

### Study population and study design

Data was collected from the Ndlovu Cohort Study (NCS), a prospective cohort study in Elandsdoorn, a rural township in the Moutse area, Limpopo Province, South Africa. A total of 1927 participants were included between November 2014 and August 2017, with the intention to include 50% HIV positive participants. Participants were recruited from the community and the Ndlovu Medical Center, a large HIV treatment center contracted by the South African Department of Health to provide medical services. Inclusion criteria were: age ≥18 years, able to provide written informed consent, and committed to long-term follow-up. Ethical approval for this study was obtained from the Human Research Ethics Committee at the University of Pretoria, Pretoria, South Africa, and the Limpopo Department of Health Ethics Committee. The methods of the NCS have been described in detail by Vos, et al. [13].

### Measurements

#### General characteristics and CV risk factors

Data was collected on age, sex, demographics, general health, HIV status, and chronic medication use including medication for hypertension and diabetes.

Information on cardiovascular (CV) risk factors and smoking was obtained with a modified version of the World Health Organization (WHO) STEPS instrument [14]. Physical activity was assessed using the International Physical Activity Questionnaire (IPAQ) on cycling and walking, moderate and vigorous activity during working, and vigorous insensitive sporting [15]. Information on diet and food security was obtained by use of the South African National Health and Nutrition Examination Survey (SANHANES) questions [16]. Information on employment and income was obtained with the National Income Dynamics Study [NIDS] Wave 3 2012 Adults Questionnaire [17]. The lower and upper bound poverty lines in 2015 were defined as 648 and 992 South African Rand (ZAR) per person per month, as defined by Statisistics of South Africa [18]. The average exchange rate in 2015 for 100 ZAR was equivalent to 7.88 United States dollars [19].

Blood pressure was measured after 5 minutes of rest in seated position with a sphygmomanometer device, and repeated on the side with the highest value. For systolic and diastolic blood pressure the average of the second and third measurement was used for analysis.

#### Biological material

Blood was collected in all participants for a non-fasting lipid profile and random glucose.

#### HIV testing

Participants with a HIV-negative status or participants with an unknown HIV status were tested for HIV at time of enrolment. Testing was performed in accordance with the South African Department of Health guideline. For HIV-positive participants, documentation was needed of a positive HIV test, or proof of being on ART, if not available, the participant was retested.

#### Carotid intima-media thickness measurements

Ultrasound measurements were obtained for all participants. A total of six images were obtained including three angles of the right (150, 120 and 90 degrees) and left (210, 240, 270 degrees) using a Meijer Arc. Images were read offline using the semi-automatically Artery Measurement System (AMS) software. The far and near wall were measured in each image at one cm distal to the tip flow divider. If this area was not clear, measurements were obtained until two cm distal of the tip flow divider. Ideally, each measurement covered a length of 10 mm; if this was not possible, a smaller segment was read. Mean CIMT was measured on each angle. For each participant the mean of all available CIMT scans was calculated.

The scans were analysed by three readers, who were blinded for all participant characteristics. The intraclass correlation coefficient to assess inter reader variability was excellent, ranging between 0.92 and 0.93 [20].

#### Cardiovascular health definition

Table 1 describes the CVD risk factors that are included in the CVHI and defines poor health, intermediate health or good health for each CVD factor, as utilized in this study.

**Table 1:** Definitions and categories of the Ideal Cardiovascular Heart Index. (BMI, body mass index; SBP, systolic blood pressure; DBP, diastolic blood pressure; * fruit and vegetable intake).

	Poor health	Intermediate health	Good health

Smoking	Yes	Former smoker, quit weeks or months ago	Never smoker or quit years ago
BMI	≥30 kg/m^2^	25.0–29.9 kg/m^2^	<25 kg/m^2^
Physical activity	None	1–149 min/week moderate intensity or 1–74 min/week vigorous intensity or 1–149 min/week combined activity	≥150 min/week moderate intensity or ≥75 min/week vigorous intensity or ≥150 min/week combined activity
Blood pressure	SBP ≥ 140 mmHgand/or DBP ≥ 90 mmHg	SBP 120–139 or DBP 80–89 mmHg or treated to goal	SBP < 120 and DBP < 80 mmHg
Glucose	≥126 mg/dL	100–125 mg/dL or treated to goal	<100 mg/dL
Cholesterol	≥240 mg/dL	200–239 mg/dL or treated to goal	<200 mg/dL
Diet*	<2 servings/day	2–4 servings/day	≥5 servings/day

The criteria that we used to define good, intermediate and poor health for smoking, glucose, cholesterol, and diet differ slightly from the AHA criteria. The AHA defines a former smoker as someone who quit smoking more than 12 months ago but our data only allowed us to categorize former smoking in weeks, months or years. In addition, random glucose was measured in the study population, instead of fasting glucose. No cholesterol lowering medication was reported among participants, as expected from South African literature [21].

The AHA definition of diet is based on five dietary components, consisting of; fruits and vegetables (≥4.5 cups/day), fish (≥ two 100 g servings/week), fiber rich whole grains (≥ three 30 g servings consisting 1.1 g of fiber per 10 g of carbohydrate/day), sodium (<1500 mg/day), and sugar sweetened beverages (≤450 kcal/week). As in the NCS only data on fruit and vegetable intake in servings/day was collected, the total servings of fruit and vegetables/day were used as a proxy for the dietary component. The cut-off for the number of servings to define CVH was based on the WHO recommendation of at least 400 grams of vegetables and/or fruits per day [22]. Poor health was assigned in the case of <2 servings/day, following the SANHANES guidelines [23], consequently intermediate health was defined as 2–4 servings/day.

### Data analysis

In total 43.3% of the participants had missing data for blood pressure measurement as the measurements in these participants were taken with a non-validated wrist device instead of the indicated arm device in the years 2016 and 2017. Besides, CIMT was missing for 7.9% of the participants due to unreadable or missing scans; data on income, and fruit and vegetables intake was missing for respectable 5.3% due to unreadable or missing scans, and for 2.1% of the participants due to incomplete questionnaires. All other variables had less than 2% missing values. Missing values were assumed to be missing at random.

Multiple imputation was performed using the ‘mice’ package of R, generating 20 imputed datasets. All valid blood pressure measurements, age, gender, HIV positivity, smoking, BMI, total cholesterol levels, glucose levels, physical activity, fruit and vegetable intake, medication use, education, and employment were included in the imputation model. Passive imputation was used for variables which needed recoding, calculations or indices for analysis. Convergence plots confirmed that the MICE algorithm had converted. Each parameter of interest was estimated in each imputed dataset separately, and combined using Rubin’s rules for analysis. Data for summarization of study characteristics in graphs were obtained by computing the mean or selecting the most likely imputed value for those variables of interest [22]. In addition, a complete case analysis was performed.

First, study characteristics were reported by sex before performing multiple imputation. For continuous data mean with standard deviation (SD) were used for normally distributed data and median with minimum and maximum for skewed data. Categorical outcomes were presented as numbers with percentage. After multiple imputation a cross-sectional analysis was performed on the data. For all CVHI factors the frequency of good, intermediate and poor health was described by sex, using the definitions of Table 1. Subsequently, the overall CVHI score (CVHIs) was calculated, based on the number of CVHI factors categorized as ‘good health.’ The overall CVHIs was divided in three categories: good (5–7 factors), intermediate (3–4 factors) and poor (0–2 factors) CVHIs. These cut-off values were chosen as they are frequently used in other publications on the CVHI, making comparisons to results from other populations possible. Next, uni- and multi-variable logistic regression analysis were carried out to assess the association of age, sex, HIV status and SES with good CVH. SES included: education (none or primary versus secondary or matric [final year 12 of formal schooling], college or university), employment (unemployed versus employed and students), and income per month (<648 versus 648–992, >992 ZAR). Model selection of the multivariable logistic regression was based on backward step selection with p-value of 0.10, after taking all above mentioned determinants into account. Odds Ratio’s (OR’s) were given as a measure of association with their 95% confidence intervals (CI).

Finally, the relation between CVHIs and CIMT was assessed. Mean CCA-CIMT was displayed per CHVIs category whilst accounting for age categories (<35, 35–54 and ≥55 years) and sex. For statistical analysis R version 3.6.1 was used [24]. A two-sided p-value below 0.05 was considered to be statistical significant.

## Results

A total of 1927 participants were included in this study, of which 887 were HIV-positive (46.0%). The mean age of the study population was 38.7 years (SD ± 12.8) (Table 2), and 1019 (56%) participants were women. Most participants completed secondary school or matric, were unemployed and had an income below the lower bound of the South African poverty line of 648 ZAR per month.

**Table 2:** Baseline characteristics of the study population by sex, before multiple imputation was conducted. (BMI, body mass index; CIMT, carotid intima-media thickness; CVD, cardiovascular disease; HIV, human immunodeficiency virus; SD, standard deviation; ZAR, South African Rand).

	Women (n = 1056)	Men (n = 871)	Missing (%)

Age in years (median [Min, Max])	38.16 [18,88]	39.44 [18,68]	0.0
HIV positivity (n, %)	529 (50.1)	358 (41.1)	0.0
Smoking (n, %)	183 (17.3)	604 (69.3)	0.0
BMI (n, %)			0.2
Underweight	101 (9.6)	191 (22.0)	
Normal	416 (39.4)	518 (59.6)	
Overweight	267 (25.3)	106 (12.2)	
Obese	271 (25.7)	54 (6.2)	
Glucose in mg/dL (mean, SD)	91.0 (54.8)	86.7 (25.9)	0.8
Total cholesterol in mg/dL (mean, SD)	167.6 (40.2)	157.5 (36.7)	0.9
Average systolic blood pressure (mmHg; mean, SD)	116.45 (23.79)	121.09 (22.08)	43.3
Average diastolic blood pressure (mmHg; mean, SD)	74.49 (13.73)	75.19 (14.10)	43.3
Moderate physical activity in min/week (mean, SD)	496.26 (729.58)	586.68 (739.58)	1.6
Vigorous physical activity in min/week (mean, SD)	99.79 (417.27)	202.64 (522.79)	1.5
Fruit and vegetable intake in servings/day (mean, SD)	1.91 (1.65)	1.93 (1.51)	2.1
Family history of CVD (n, %)	183 (17.3)	98 (11.3)	0.2
Educational attainment (n, %)			0.0
None or primary	231 (21.9)	229 (26.3)	
Secondary or matric	724 (68.6)	576 (66.1)	
College or university	101 (9.6)	66 (7.6)	
Employment (n, %)			0.0
Employed	182 (17.2)	215 (24.7)	
Unemployed	807 (76.4)	574 (65.9)	
Student	67 (6.3)	82 (9.4)	
Income per person per month in ZAR (mean, SD)			5.3
<648	688 (66.6)	455 (57.5)	
648–992	90 (8.7)	54 (6.8)	
>992	255 (24.7)	282 (35.7)	
Relationship status (n, %)			0.0
Married or long term relationship	603 (57.1)	520 (59.7)	
Single	367 (34.8)	279 (32.0)	
Widowed	60 (5.7)	30 (3.4)	
Other	26 (2.5)	42 (4.8)	
Mean CIMT in mm (mean, SD)	0.60 (0.11)	0.63 (0.14)	7.9

**Figure 1:**
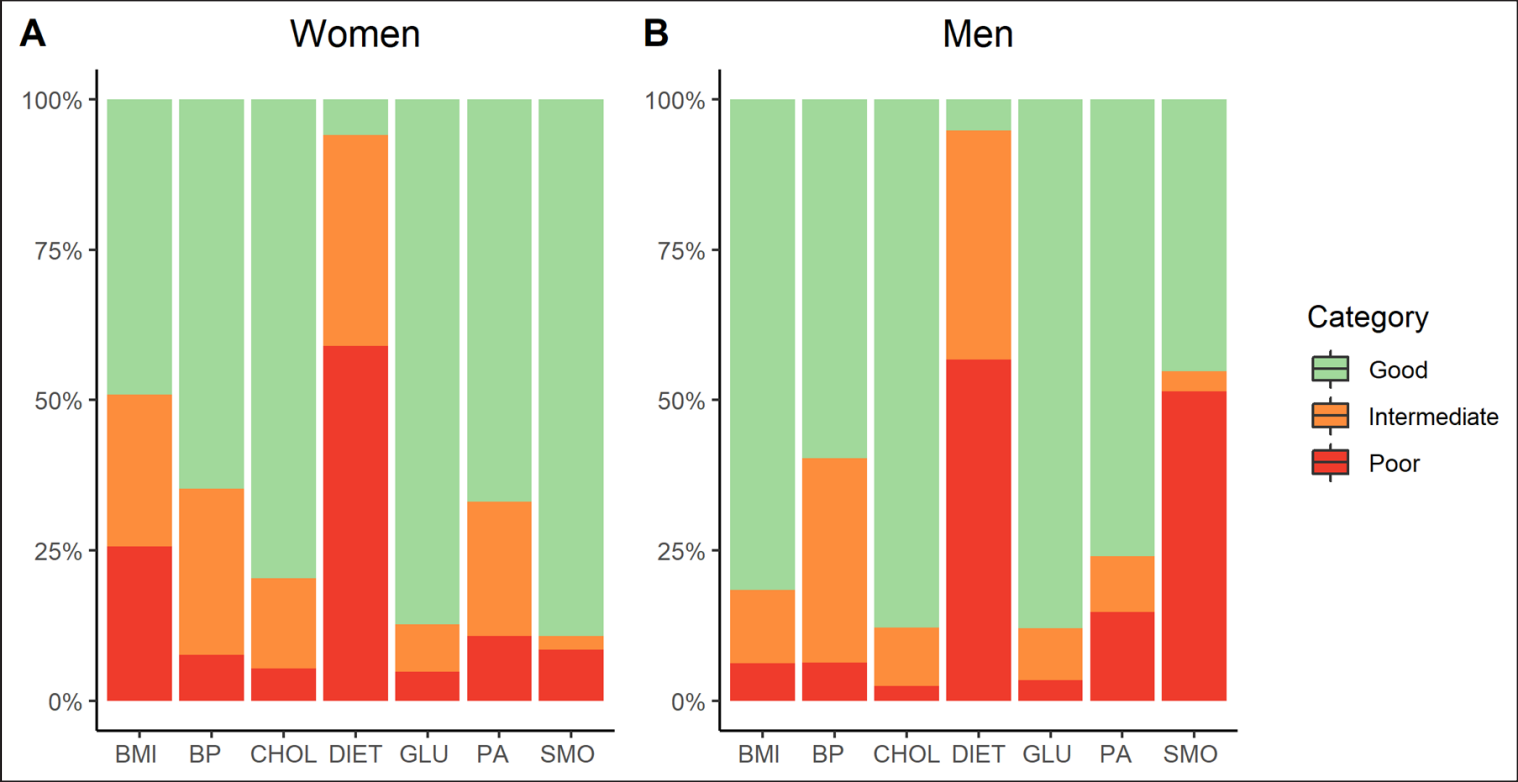
The prevalence of poor, intermediate, and good cardiovascular health for each factor of the cardiovascular health score, by sex.

For all factors of the CVHI, the percentages for poor, intermediate and good CVH are shown in Figure 1. Diet contributed least to a good CVHIs as less than 6% of the population managed to have the recommended intake of at least five servings of fruit and vegetables a day. Men were six times more likely to smoke, whereas women were almost three times more likely to be overweight. The majority of the study population had a good contribution to the CVHIs for physical activity, cholesterol, and glucose, with respectively 71%, 83% and 88% of the participants reaching good CVH.

A total of 62% of all participants were classified as having adequate blood pressure measurement. Before imputation, this percentage was 56% among those 1122 participants with adequate blood pressure measurements. HIV positivity was almost twice as high, and the mean number of factors classifying for good health was with 3.84 (SD ± 0.91) out of six higher than those with blood pressure measurements (3.78 (SD ± 1.00)). Lower glucose levels and a healthier BMI contributed to this higher score; smoking had a negative contribution to this score.

**Figure 2:**
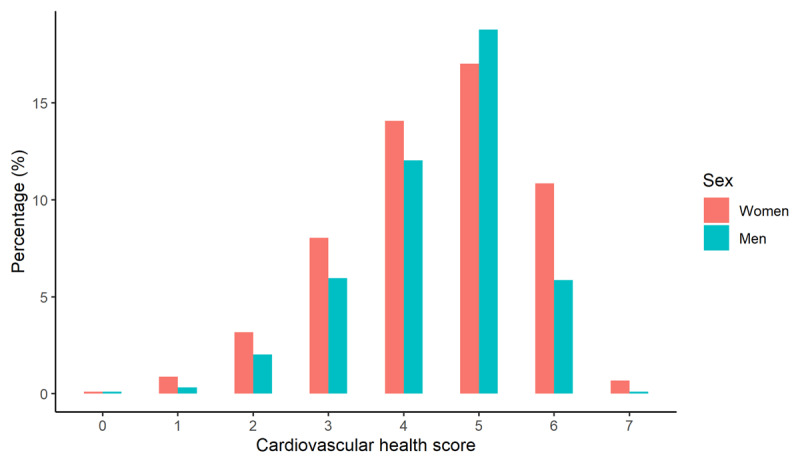
Distribution of the cardiovascular health score (CVHIs) by sex. CVHIs is constructed by counting the number of favorable scoring risk factors, including: smoking, body-mass index, physical activity, blood pressure, glucose, total cholesterol, and diet.

The average CVHIs was 4.4 (SD ± 1.2). A total of 53% of the participants were classified as having good CVH according to the CVH categorization, with intermediate health (40%), followed by poor health (7%) (Figure 2). Less than 1% of the overall population had a good score on all seven factors. After complete case analysis, 57% of the participants reached a good CVH conform to the CVH categorization.

Univariable logistic regression analysis showed a significant association between good CVH and younger age, higher educational attainment, and being a student. Following multivariable regression, younger age, and being a student remained significantly associated with good CVH. HIV positivity became significantly associated with good CVH in the multivariable analysis (Table 3). These results were similar for completed case analysis (Supplementary File 1).

**Table 3:** Logistic regression estimates for a good cardiovascular health score (CVHIs) of at least 5, for age, sex, HIV-status, education, employment and income. Adjusted odds ratio’s (OR’s) are adjusted for age, HIV-status, education and employment. (HIV, human immunodeficiency virus; OR, odds ratio; ZAR, South African rand).

		Crude OR (95% CI)	Adjusted OR (95% CI)

**Age (years)**	<35	Reference	Reference
	35–54	0.33 [0.27;0.42]	0.76 [0.72;0.81]
	≥55	0.15 [0.11;0.22]	0.66 [0.62;0.72]
**Male sex**		1.04 [0.85;1.28]	–
**HIV-positive**		1.14 [0.93;1.40]	1.14 [1.08;1.19]
**Education**	None or primary	Reference	
	Secondary or metric	2.52 [1.95;3.25]	–
	College or university	2.49 [1.67;3.69]	
**Employment**	Employed	Reference	Reference
	Unemployed	1.01 [0.78;1.29]	0.98 [0.93;1.04]
	Student	5.67 [3.38;9.52]	1.24 [1.12;1.37]
**Income (ZAR)**	<648	Reference	–
	648–992	1.15 [0.79;1.69]	
	>992	1.02 [0.82;1.27]	

### Association of CVHIs with CIMT

**Figure 3:**
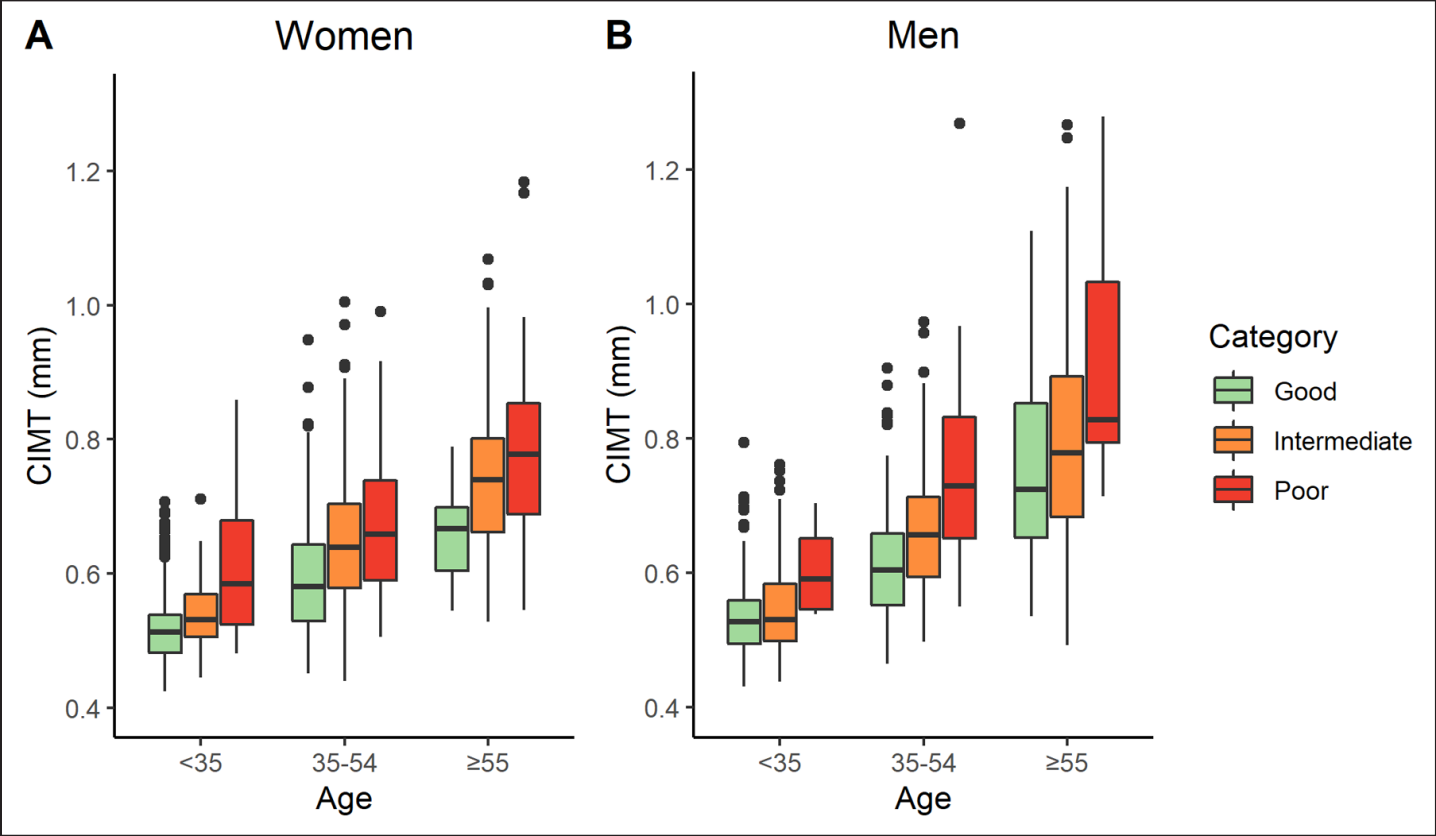
Mean CIMT (mm) by category based on the cardiovascular health score (CVHIs) and sex, where CIMT is the carotid intima-media thickness.

Boxplots of mean CIMT by sex, CVH and age are shown in Figure 3. Mean CIMT was inversely related to CVHIs and positively to age, indicating that CIMT increases with adverse CV risk factors and CIMT increases with age.

## Discussion

### Key findings

Using CVHI, over half of this rural South African population had a good CVHIs. Determinants that were associated with good CVHIs were younger age, being a student, and HIV positivity. CVHIs was directly related to CIMT.

### Discussion of key findings

A recent meta-analysis including papers published between 2010 and 2018, reported a global estimate of good CVH of 19.6% (15.2%–23.9%, 95% CI), and, for those younger than 60 years, an estimated good CVH of 25.8% [5]. However, only one study from Africa was included (Uganda), reporting good CVH for 58.6% (51.1–65.0, 95% CI) of the population [5]. A recent study conducted in rural Ghana, found a good CVH for 60.8% of the population [7]. The estimate of good CVH of 53% as found in the current analysis is lower than these estimates, but considerably higher than the global estimate found in the meta-analysis. With a mean age of 38 years the NCS is a relatively young population, representative of the Moutse area, a typical rural area in South Africa. The on average younger age of our study population might explain the better CVH as compared to the estimate reported in the meta-analysis. This does, however, not explain the differences between the Ghanaian and Ugandan study, and our study; as the populations included in these studies were older with a mean age of respectively 45 and 50 years. The differences may represent a shift within South Africa, including in rural areas, towards a western lifestyle with less availability of vegetables and fruits (supported by our data), easier access to convenience food, and decreased physical activity in comparison to less transited populations [25].

Normal glucose and cholesterol measurements, followed by physical activity and blood pressure, contributed to a good CVHIs. This is not surprising as our population is still relatively young [26]. Diet negatively contributed to CVHIs, with only 5.6% of the population obtaining good CVH for fruit and vegetable intake. Poverty, observed to be highly prevalent in the rural study population, as well as lack of availability of fresh food and vegetables [25], may limit the possibility to make healthy choices [27].

The higher proportion of good CVH might be explained by the slightly higher CVHIs, and larger proportion of HIV positivity as is observed in our analysis.

Sex-dependent risk factors in this study were BMI and smoking. Men were far more likely to be a current smoker, and women were almost three-fold more likely to be overweight. These results are in line with patterns observed by van Nieuwenhuizen, et al. [7], in a study undertaken in Ghana, and supported by the meta-analysis by Peng, et al. [5]. On the contrary, no sex-dependent association with BMI was observed in these studies [5].

HIV-positive participants were observed to have a better CVH, results that are in line with the study by Martin, et al. undertaken in Uganda [6], and could in part be attributed to more frequent access to health care, increased focus on a healthy lifestyle and management of HIV-related non-communicable conditions. However, a lower CVD risk profile in the context of HIV infection might not translate to less CVD events. Immune system activation and inflammatory responses likely play a role in CVD development; several studies have indicated that an HIV infection might facilitate the development and progression of atherosclerosis. Exact mechanisms underlying HIV mediated atherosclerosis have not yet unravelled and require further research [28,29].

In accordance with earlier studies, CIMT measurements related well with the CVHIs [6,30]. This suggests that CVHIs could be used as an easier proxy for CIMT. Several studies have identified CIMT as a marker to improve risk prediction for future CVD [31,32]. Longitudinal studies assessing CVD outcome data are needed to investigate how well CVHIs predicts the occurrence of CVD events.

The individual risk factors composing the CVHIs, are not weighted based on their individual contribution to future CVD events, and are therefore not ideal for CVD risk prediction. Nonetheless, the use of CVHIs and individual factors of the CVHI are suitable for assessing current CV health status as undertaken in our study and for monitoring changes over time.

### Limitations

There are some limitations that need mentioning. Adjustments were made to the CVHI definitions as proposed by the AHA, based on data availability. Fruit and vegetable intake were used as a proxy for five dietary components, in line with other studies using the AHA definition. Measurement of random glucose instead of fasting glucose might have led to a slight underestimation of good CVH.

Around 15% of our participants were underweight. Underweight is acknowledged as a health risk by the AHA, but as it is a relatively rare condition in high-income countries, it was not integrated in the original CVHI recommendation [4]. This might have led to an overestimation of ‘good CVH’ for BMI.

Almost half of study participants had missing data on at least one variable; blood pressure was with 43.3% the largest contributor. Multiple imputation was conducted to avoid possible bias due to missing data. With missingness at random, population estimates are likely to be more precise and valid as opposed to complete case analysis. The proportion of participants with a desired blood pressure measurements was larger among those with missing data. This is most likely due to the higher percentage of HIV positivity in those with missing blood pressure data, being associated with lower blood pressure. No changes in the average CHVS is observed after imputation. Although multiple imputation strives towards the most likely values, complete data on all participants would have been most accurate.

By design, half of the included participants in the Ndlovu Cohort Study (NCS) were HIV infected. This percentage is higher than the HIV prevalence of 16.8% among adults between 18 and 49 in South Africa in 2014 when NCS recruitment commenced [33]. The oversampling of HIV positive participants, might have led to a more favourable CVH estimate in our study, as HIV status was associated with a more favourable CVH score.

For the current cross-sectional analysis of NCS baseline data, CIMT was used as a marker of subclinical atherosclerosis and proxy for cardiovascular risk [34]. The value of CIMT to predict future cardiovascular events in daily practice is not universally recognized [35], however CIMT provides in the absence of cardiovascular event data a valuable proxy of future cardiovascular risk in our and similar settings.

## Conclusion

In this rural sub-Saharan setting, more than half of the population showed a good CVHIs. Remedial actions to further improve health include smoking reduction especially in men, better access and affordability of nutritious and healthy food, and continuing to encourage physical activity. Interventions should be adapted to local situations, informing about affordable and healthier options. The CVHIs was related with CIMT; indicating that CVHIs could be used as a surrogate marker for CVD.

## Abbreviations

AHA = American Heart Association, AMS = Artery Measurement System, CIMT = carotid intima-media thickness; CVH = cardiovascular health, CVHI = cardiovascular health index; CVHIs = cardiovascular index health score, IPAQ = International Physical Activity Questionnaire, NIDS = National Income Dynamics Study, SANHANES = South African National Health and Nutrition Examination Survey, SSA = sub-Saharan Africa, ZAR = South African rand.
